# Artificial Intelligence in Undergraduate Medical Education: A Cross-Sectional Study of Utilization Patterns and Perceptions Among Medical Students

**DOI:** 10.7759/cureus.105503

**Published:** 2026-03-19

**Authors:** Raju R Bokan, Rashmi Malhotra, Mukund Vatsa, Kanchan Bisht, Mukesh Singla, Rajeev Choudhary

**Affiliations:** 1 Anatomy, All India Institute of Medical Sciences, Rishikesh, Rishikesh, IND

**Keywords:** artificial intelligence, chatgpt, cross-sectional study, machine learning algorithms, medical education, medical students, undergraduate mbbs

## Abstract

Background

Artificial intelligence (AI), particularly tools such as ChatGPT, has quickly become part of how medical students study and revise. In day-to-day academic settings, many students are already using AI to clarify concepts and prepare for exams. However, there are limited data from Indian medical institutions regarding how frequently these tools are used and how students perceive them.

Objective

To assess awareness, patterns of use, perceived usefulness, reliability, and concerns related to AI tools among undergraduate medical students at All India Institute of Medical Sciences (AIIMS) Rishikesh.

Methods

A cross-sectional questionnaire-based study was conducted among 297 undergraduate MBBS students at AIIMS, Rishikesh. The study was designed and carried out by faculty from the Department of Anatomy. A structured survey collected information on the frequency of AI use, preferred platforms, academic applications, trust in AI-generated information, and perceived risks. Data were analyzed using descriptive statistics, and chi-square tests were applied to assess associations between selected variables.

Results

Most students (91.6%) reported using AI tools for academic purposes, with ChatGPT (OpenAI, San Francisco, California, US) being the most commonly used platform (96%). The majority used AI to better understand difficult topics (88.2%). Although 70.7% considered AI outputs to be good or reliable, concerns were common, particularly regarding accuracy (72.1%) and data privacy (50.2%). Students who used AI more frequently were significantly more likely to support formal integration of AI into the medical curriculum (χ² = 16.82, p = 0.001). Overall, 69.4% favored structured incorporation of AI training.

Conclusion

AI tools are already widely used by undergraduate medical students and are largely viewed as helpful supplementary learning resources. At the same time, students remain cautious about accuracy and ethical implications. These findings suggest that rather than ignoring AI use, medical institutions should consider structured guidance and training to ensure responsible and effective integration.

## Introduction

Artificial intelligence (AI) has increasingly become part of healthcare systems and medical education worldwide. Advances in computational modeling and machine learning have enabled the development of systems capable of simulating aspects of human reasoning and decision-making [1]. Over time, improvements in natural language processing have allowed AI tools to generate structured, context-aware responses to complex queries, expanding their utility in academic environments [2,3]. More recently, generative AI models have become widely accessible, further accelerating their integration into education [4]. In medical education, AI has been explored in multiple domains, including adaptive learning systems, automated assessment, clinical simulation, and competency tracking [5,6]. Intelligent virtual case systems and AI-assisted interpretation tools have also demonstrated potential in enhancing diagnostic reasoning and structured learning experiences [7]. With the emergence of large language models, such as ChatGPT (OpenAI, San Francisco, California, US), attention has shifted toward conversational AI systems capable of summarizing medical content, generating explanations, and assisting with examination preparation. Several recent studies have evaluated the performance of large language models on standardized medical licensing examinations. Investigations assessing ChatGPT’s performance on the United States Medical Licensing Examination (USMLE) demonstrated performance at or near passing thresholds in selected components [8]. Subsequent comparisons between different model versions, including GPT-3.5 and GPT-4, reported improvements in reasoning accuracy and structured response generation [9,10]. Similar evaluations in European and specialty examinations have further highlighted both the strengths and limitations of these systems [11]. Despite encouraging performance data, important concerns remain. The need for regulatory oversight of large language models in healthcare has been emphasized in recent literature [12]. While AI-assisted educational tools may enhance accessibility and efficiency, questions regarding reliability, contextual understanding, and the potential for confidently generated inaccuracies persist [13,14]. Ethical considerations, including bias, transparency, and responsible academic use, have also been actively discussed [15]. In parallel, efforts to improve AI literacy among medical students have begun to emerge. Surveys assessing medical students’ knowledge and attitudes toward AI have demonstrated variable awareness and preparedness [16]. Educational interventions, including structured AI modules and flipped classroom approaches, have shown promise in improving student confidence and conceptual understanding [17,18]. However, broader questions regarding the limits of AI in medical specialization and its long-term educational impact continue to be explored [19]. Although international literature has expanded rapidly, institutional-level data examining real-world utilization patterns among undergraduate medical students in India remain limited. Understanding how frequently students use AI tools, how they perceive their reliability, and what concerns they hold is essential before structured curricular integration is considered.

The present study was therefore conducted at AIIMS Rishikesh to evaluate utilization patterns, perceptions, and concerns related to artificial intelligence among undergraduate medical students. By generating institution-specific data, this study aims to contribute evidence that may inform thoughtful and responsible integration of AI into undergraduate medical education.

## Materials and methods

Study design and setting

This cross-sectional questionnaire-based study was conducted in the Department of Anatomy at the All India Institute of Medical Sciences (AIIMS), Rishikesh, Uttarakhand, India. The study was designed to evaluate utilization patterns, perceptions, and concerns related to AI tools among undergraduate medical students.

Participants

The study population included undergraduate MBBS students enrolled at AIIMS Rishikesh. Participation was voluntary. A total of 297 students completed the survey and were included in the final analysis. No patients or clinical subjects were involved.

Data Collection

Data were collected using a structured, self-administered questionnaire designed to assess the awareness and frequency of AI use, preferred AI platforms, academic applications, perceived reliability, and concerns regarding AI-generated information. The questionnaire included multiple-choice and Likert-scale items. The survey was distributed electronically, and responses were recorded anonymously. No identifiable personal information was collected. Participation implied informed consent.

Statistical Analysis

Descriptive statistics were used to summarize demographic characteristics and survey responses. Categorical variables were expressed as frequencies and percentages. Associations between selected variables, including 2 of 9, frequency of AI use, and support for curriculum integration, were evaluated using the chi-square test. A p value of less than 0.05 was considered statistically significant. Statistical analysis was performed using standard statistical software.

Ethical Considerations

The study involved voluntary participation of undergraduate medical students and did not involve patients, clinical interventions, biological samples, or identifiable personal data. The research activity was conducted as a minimal-risk educational survey within the scope of routine academic practice.

## Results

Demographic characteristics

A total of 297 undergraduate MBBS students participated in the study. Among them, 65% were male and 35% were female. Students from all professional years were represented, with the majority belonging to the third professional year (64.3%). The detailed demographic distribution is presented in Table 1.

**Table 1:** Demographic characteristics of participants (N = 297) This table summarizes the gender distribution and academic year of the participants included in the cross-sectional survey conducted at AIIMS Rishikesh.

Variable	Category	Frequency (n)	Percentage (%)
Gender	Male	193	65
	Female	104	35
Year of Study	I Prof MBBS	32	10.8
	II Prof MBBS	53	17.8
	III Prof MBBS	191	64.3
	Final Prof MBBS	16	5.4
	Intern	5	1.7

AI utilization patterns

Most participants (91.6%) reported using AI tools for academic purposes. Weekly use was the most common frequency (56.9%), followed by monthly use (25.3%) and daily use (12.5%). ChatGPT was the most frequently used platform (96%), followed by Gemini (55.6%) and Perplexity (28.3%). The distribution of AI usage patterns is summarized in Table 2. The primary reason for AI use was clarification of difficult concepts (88.2%). Other uses included accessing medical literature (38.4%), creating presentations (35%), conducting quizzes (22.9%), and simulation-based learning (21.5%).

**Table 2:** AI usage patterns among undergraduate medical students This table presents the prevalence of AI tool usage, frequency of use, and the most commonly used AI platforms among study participants.

Parameter	Category	Frequency (n)	Percentage (%)
Ever Used AI	Yes	272	91.6
	No	25	8.4
Frequency of Use	Daily	37	12.5
	Weekly	169	56.9
	Monthly	75	25.3
	Rarely	16	5.3
Most Used Platform	ChatGPT	285	96
	Gemini	165	55.6
	Perplexity	84	28.3

Perceptions and reliability

A majority of students (76.5%) agreed or strongly agreed that AI tools help in understanding difficult concepts. Approximately 70.7% rated AI outputs as good and reliable, while 9.8% rated them as excellent. Only a small proportion (5.1%) considered AI outputs to be unreliable. Most students reported that AI tools were easy to use (89.9%). Trust in AI-generated information was moderate, with 55.9% expressing agreement and a substantial proportion remaining neutral. Perceptions regarding usefulness and reliability are summarized in Table 3.

**Table 3:** Perceptions and concerns regarding AI use in medical education This table summarizes student-reported perceptions of AI effectiveness, support for curriculum integration, and major concerns related to accuracy, privacy, and professional impact.

Variable	Category/Response	Percentage (%)
AI Helps Understand Concepts	Agree/Strongly Agree	76.5
AI Rated Good or Excellent	—	80.5
Support Curriculum Integration	Yes	69.4
Major Concern: Accuracy	—	72.1
Major Concern: Data Privacy	—	50.2
Believe AI Will Assist (Not Replace)	—	47.8

Concerns regarding AI

Despite widespread use, concerns were frequently reported. The most common concern was accuracy of content (72.1%), followed by data privacy (50.2%), risk of errors leading to adverse outcomes (49.8%), and potential replacement of physicians (42.8%). Ethical concerns were reported by 30% of participants.

Curriculum integration

A majority of students (69.4%) supported formal integration of AI training into the undergraduate medical curriculum, while 12.5% opposed integration and 20.5% were uncertain.

Inferential analysis

Chi-square analysis demonstrated a statistically significant association between frequency of AI usage and support for curriculum integration (χ² = 16.82, p = 0.001). Students who reported more frequent AI use were significantly more likely to support structured incorporation of AI training. No statistically significant association was observed between gender and trust in AI reliability (p > 0.05).

## Discussion

The present cross-sectional study conducted at AIIMS Rishikesh demonstrates widespread adoption of AI tools among undergraduate medical students, with more than 90% reporting active use. This high prevalence reflects a broader global shift toward AI-enabled academic support systems. Early conceptual frameworks describing AI in healthcare education emphasized its potential to simulate cognitive processes, enhance pattern recognition, and facilitate adaptive learning environments [1,2]. Advances in natural language processing and conversational AI systems have since expanded these capabilities, allowing real-time interactive engagement with complex biomedical content [3,4].

Systematic analyses of AI applications in medical education have highlighted diverse use cases, including automated assessment, intelligent tutoring systems, predictive analytics, and virtual patient simulations [5-7]. These reviews suggest that AI is no longer experimental but increasingly integrated into formal and informal learning workflows. Our findings align with this trajectory, as the majority of students reported using AI tools weekly or more frequently, primarily for concept clarification.

Recent investigations evaluating large language models, such as ChatGPT, have further accelerated interest in AI within academic medicine. Performance studies assessing ChatGPT on the United States Medical Licensing Examination (USMLE) demonstrated that the model achieved scores at or near passing thresholds in several components [8]. Subsequent comparative evaluations between GPT-3.5 and GPT-4 revealed improved reasoning accuracy and structured clinical responses in the newer architecture [9,10]. Similar assessments in European specialty examinations reported variable but notable performance across domains [11]. These findings likely contribute to the confidence students place in AI tools for academic reinforcement.

However, performance studies consistently emphasize variability across question types and specialties [12]. While LLMs perform well in text-based reasoning tasks, limitations are evident in image-based interpretation, contextual judgment, and nuanced ethical reasoning [13,14]. This heterogeneity parallels our data: although a majority rated AI outputs as good or reliable, a similarly high proportion expressed concern regarding accuracy. The coexistence of trust and skepticism reflects a pragmatic student perspective: AI is useful but not infallible.

Beyond examination performance, AI has been evaluated as a tool for generating illness scripts and multiple-choice questions. Studies have shown that GPT-4 can generate clinically coherent illness scripts and draft examination questions that require minimal faculty revision [15,16]. In controlled comparisons, AI-generated questions were judged comparable in quality to human-authored items after expert review [17]. These findings resonate with our respondents’ use of AI for preparing presentations and quizzes. Nevertheless, the literature uniformly recommends human oversight to prevent propagation of inaccuracies [18].

AI-assisted simulation and intelligent tutoring systems have also been explored extensively. Randomized and quasi-experimental studies indicate that AI-driven adaptive tutoring can improve diagnostic reasoning and examination performance when compared to traditional didactic approaches [19,20]. Virtual standardized patients powered by AI have demonstrated enhanced learner engagement and immediate feedback capabilities [21]. Furthermore, machine learning-based analytics have been used to track learner progression and identify performance gaps in competency-based education models [22,23]. These structured implementations provide empirical support for our students’ perception that AI improves conceptual clarity.

Despite encouraging outcomes, methodological limitations remain common in AI education research. Several studies report small sample sizes, single-center designs, and reliance on self-reported outcomes rather than objective performance metrics [24,25]. Our study shares some of these constraints, reinforcing the need for longitudinal and multicenter investigations. Nonetheless, descriptive institutional data remain essential for informing curriculum development.

Ethical and regulatory concerns have emerged as central themes in AI discourse. Scholars have emphasized the need for governance frameworks addressing transparency, accountability, and bias mitigation [26,27]. Concerns regarding data privacy and algorithmic bias are particularly relevant in healthcare contexts, where inaccurate or biased outputs may have serious implications. In our cohort, approximately half of the students expressed concerns regarding data privacy, and nearly three-quarters were concerned about content accuracy. These findings are consistent with broader ethical analyses cautioning against uncritical adoption of generative AI systems [28].

The question of whether AI might replace educators or clinicians has also been examined. Studies comparing AI-generated educational content with human instruction generally conclude that AI functions best as an adjunct rather than a replacement [29]. While AI can efficiently generate structured explanations and draft materials, it lacks contextual judgment, empathy, and accountability, qualities central to medical professionalism. Our respondents largely echoed this sentiment, with most believing AI would assist rather than replace teachers.

Structured AI literacy programs have been proposed as a solution to bridge enthusiasm and caution. Pilot curricula incorporating AI fundamentals into medical training have demonstrated improvements in student confidence and conceptual understanding of machine learning principles [29]. These interventions emphasize critical appraisal, responsible use, and awareness of model limitations. The strong support for curriculum integration observed in our study suggests readiness for similar initiatives at our institution.

Taken together, existing literature supports a balanced interpretation of our findings. AI tools are demonstrably capable of supporting medical learning through rapid information synthesis, adaptive explanation, and automated content generation. However, concerns regarding reliability, bias, and privacy remain valid and require institutional oversight. The convergence of high utilization and cautious optimism in our cohort reflects the broader global experience documented across multiple studies [1-29].

Our data contribute institution-level evidence from an Indian tertiary academic center, adding to a literature base that has been dominated by Western institutions. Cultural, curricular, and infrastructural differences may influence AI adoption patterns, underscoring the importance of local data. As AI systems continue to evolve, institutions must develop policies that encourage responsible use while preserving academic integrity and professional standards.

In summary, comparison with existing literature demonstrates that our findings are consistent with global trends: widespread adoption, perceived academic benefit, recognition of limitations, and strong support for structured integration. The challenge moving forward lies not in deciding whether AI will influence medical education (it already does) but in ensuring that its integration is thoughtful, evidence-based, and ethically grounded.

**Figure 1:**
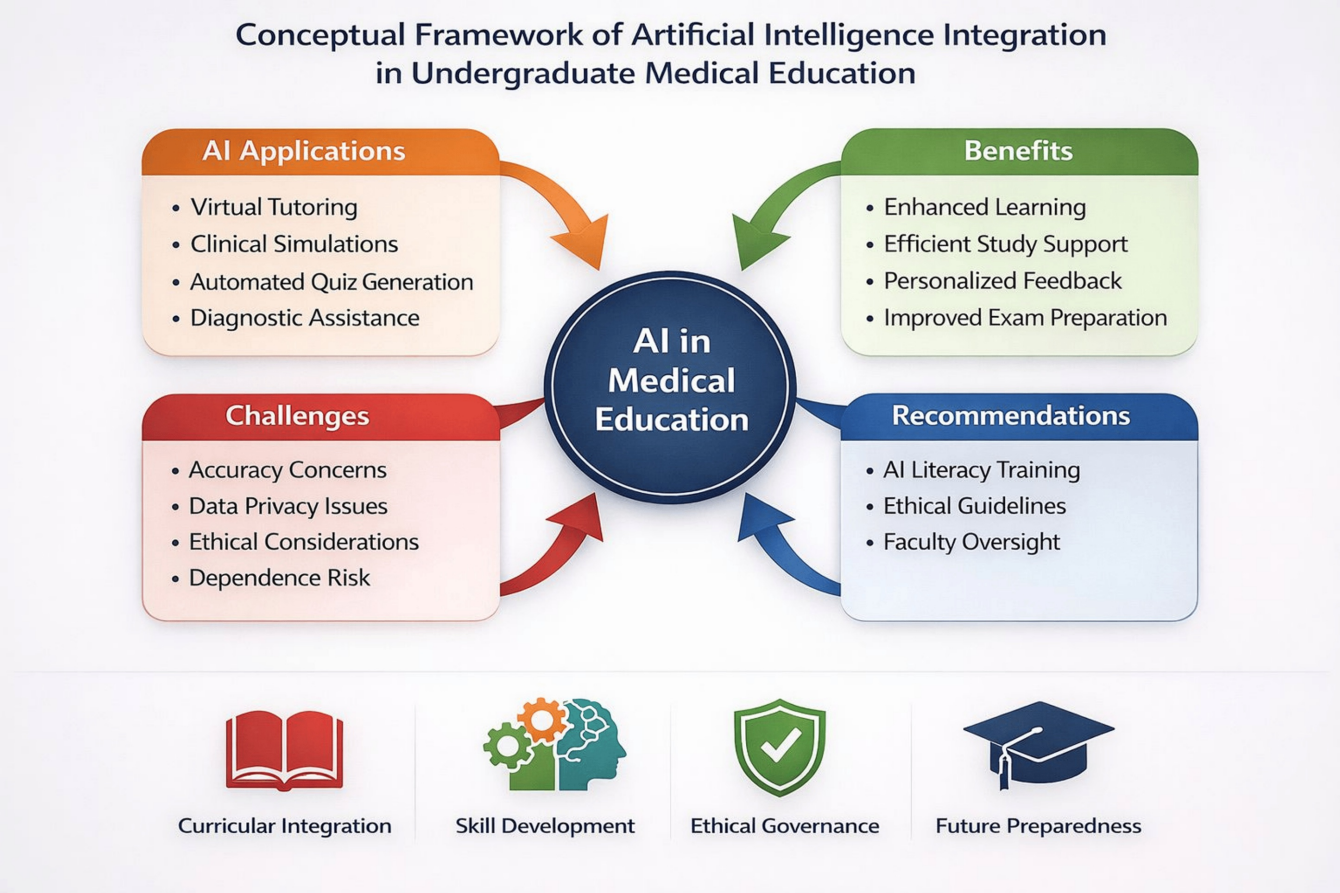
Conceptual framework for the integration of artificial intelligence in undergraduate medical education The figure illustrates a conceptual model summarizing the role of artificial intelligence (AI) in undergraduate medical education based on the findings of this study and existing literature. The framework highlights key domains of AI application, including academic support, assessment enhancement, and learning analytics; perceived benefits, such as improved conceptual clarity and accessibility; and moderating factors, including concerns related to accuracy, data privacy, ethical implications, and over-reliance. The model emphasizes the need for structured AI literacy training, institutional guidelines, and faculty oversight to ensure responsible and effective integration into medical curricula. This figure was created using Microsoft PowerPoint (Microsoft Corporation, Redmond, WA, US).

Based on the findings of this study and comparison with existing literature, a conceptual framework outlining the potential integration of artificial intelligence into undergraduate medical education is proposed (Figure 1). The framework highlights core domains of application, perceived educational benefits, and moderating factors such as accuracy concerns, ethical considerations, and the need for structured oversight.

Recent scholarship has continued to examine the role of artificial intelligence and large language models in medical education. Contemporary analyses suggest that AI-enabled learning tools can support conceptual understanding, adaptive learning, and rapid access to educational resources within modern medical curricula [30]. Reviews focusing specifically on large language models in medical education highlight both their pedagogical potential and the need for cautious integration due to concerns about reliability and hallucinated information [31]. Within radiology and imaging education, LLMs have also been discussed as tools capable of assisting learners with report structuring, differential diagnosis generation, and interpretation of imaging findings, although ethical and practical considerations remain important [32]. Experimental evaluations have further explored the capability of models such as ChatGPT, Bard, and Bing in solving radiology case vignettes, demonstrating encouraging but variable diagnostic reasoning performance [33]. Investigations evaluating AI performance in knowledge-based educational tasks have similarly shown that large language models can assist learners in answering structured medical questions aligned with competency-based curricula, although accuracy varies depending on the complexity of prompts and the depth of reasoning required [34]. Beyond examination preparation, AI tools have also been explored for generating simplified educational content and patient-education materials, demonstrating their ability to translate complex biomedical concepts into accessible explanations [35]. Scholars have also suggested that the rapid evolution of large language models may significantly influence the future structure of medical education by supporting self-directed learning and personalized knowledge acquisition [36,37]. Commentary within radiology literature further highlights both the promise and limitations of these systems, emphasizing that AI is more likely to augment rather than replace clinical expertise and professional judgment [38,39].

Additional studies within radiology have explored how large language models may assist clinicians and trainees in interpreting imaging reports and generating structured explanations for complex findings [40]. Performance-based evaluations of ChatGPT on radiology board-style examinations suggest that these systems can provide plausible reasoning pathways but still demonstrate limitations in accuracy and contextual interpretation [41]. Similarly, research investigating AI-generated differential diagnoses from imaging patterns has demonstrated feasibility while underscoring the need for human oversight in clinical decision-making [42]. Comparative analyses of multiple large language models applied to cardiovascular and thoracic imaging scenarios further illustrate variability in model performance across clinical contexts [43]. Investigations evaluating GPT-4 in radiologic decision-support tasks have also reported promising results when AI is used as an adjunct tool rather than an autonomous diagnostic system [44]. Moreover, context-specific AI chatbots designed for radiology have shown potential to outperform general-purpose models when trained on domain-specific knowledge bases [45].

Importantly, several recent surveys have specifically evaluated how medical students perceive and utilize artificial intelligence tools in educational settings. Biri et al. reported that undergraduate medical students frequently use large language models to clarify difficult concepts and supplement traditional study resources, although concerns regarding accuracy and misuse remain common [46]. Similarly, Tung and Dong found that medical students generally hold positive attitudes toward AI technologies but emphasized the importance of structured educational training to ensure responsible implementation [47]. Studies examining broader student perceptions of artificial intelligence in medical education have also documented strong interest in integrating AI literacy into medical curricula while acknowledging ethical concerns and reliability limitations [48]. Investigations focusing on students’ experiences with ChatGPT have further demonstrated widespread awareness and use of generative AI tools for academic purposes, although cautious trust in AI-generated information persists [49]. Comparable findings have been observed among radiology professionals evaluating the integration of artificial intelligence into imaging practice, highlighting both opportunities and implementation challenges [50]. Educational analyses examining AI integration within medical curricula also emphasize the need for carefully designed training frameworks that balance technological innovation with critical appraisal skills [51]. Additional commentary has further emphasized that the quality of large language model responses is highly dependent on prompt structure and user interaction, reinforcing the importance of AI literacy among learners [52]. Finally, broader discussions on revitalizing medical education through faculty development and educational innovation highlight the importance of preparing both students and educators for the evolving role of digital technologies in healthcare training [53].

Limitations

This study has several limitations. First, it was conducted at a single tertiary-care academic institution, which may limit the generalizability of the findings to other medical colleges with different curricular structures or technological exposure. Second, the study relied on self-reported data, which may be subject to response bias and social desirability bias. Third, the cross-sectional design captures perceptions and usage patterns at a single point in time and does not allow an assessment of longitudinal trends or causal relationships between AI usage and academic performance. Additionally, objective measures of learning outcomes were not evaluated, and the study did not assess the quality or accuracy of AI-generated content used by students. Future multicenter and longitudinal studies incorporating objective academic performance metrics would provide stronger evidence regarding the educational impact of AI tools.

## Conclusions

This cross-sectional study conducted at AIIMS Rishikesh demonstrates that AI tools are already widely integrated into the academic practices of undergraduate medical students. A substantial majority of students reported regular use of AI platforms, particularly for clarification of difficult concepts, literature access, and academic preparation. While most participants perceived AI outputs as helpful and generally reliable, significant concerns regarding accuracy, data privacy, and ethical implications persist. The findings suggest that AI is not viewed as a replacement for educators but rather as a supplementary academic tool. Importantly, students who used AI more frequently were significantly more likely to support formal curricular integration, indicating readiness for structured institutional engagement with these technologies.

Given the rapid evolution of AI systems and their increasing accessibility, medical institutions should consider proactive strategies for integration rather than reactive restriction. Structured AI literacy training, clear institutional guidelines, faculty development initiatives, and ethical oversight mechanisms will be essential to ensure responsible and effective incorporation into undergraduate medical education. Future research should focus on multicenter studies, objective assessment of learning outcomes associated with AI-assisted study, and longitudinal evaluation of its impact on clinical reasoning and professional development. Thoughtful implementation, guided by evidence and institutional policy, will determine whether artificial intelligence becomes a transformative adjunct or an unregulated academic shortcut in medical training.
